# Impact of protocolized fluid management on electrolyte stability in patients undergoing continuous renal replacement therapy

**DOI:** 10.3389/fmed.2022.915072

**Published:** 2022-08-31

**Authors:** Song In Baeg, Junseok Jeon, Danbee Kang, Soo Jin Na, Juhee Cho, Kyunga Kim, Jeong Hoon Yang, Chi Ryang Chung, Jung Eun Lee, Wooseong Huh, Gee Young Suh, Yoon-Goo Kim, Dae Joong Kim, Hye Ryoun Jang

**Affiliations:** ^1^Division of Nephrology, Department of Internal Medicine, Myongji Hospital, Hanyang University Medical Center, Goyang, South Korea; ^2^Division of Nephrology, Department of Medicine, Samsung Medical Center, Samsung Biomedical Research Institute, Sungkyunkwan University School of Medicine, Seoul, South Korea; ^3^Department of Clinical Research Design and Evaluation, Samsung Advanced Institute for Health Sciences and Technology, Sungkyunkwan University, Seoul, South Korea; ^4^Center of Clinical Epidemiology, Samsung Medical Center, Seoul, South Korea; ^5^Department of Critical Care Medicine, Samsung Medical Center, Sungkyunkwan University School of Medicine, Seoul, South Korea; ^6^Statistics and Data Center, Samsung Medical Center, Seoul, South Korea

**Keywords:** acute kidney injury, continuous renal replacement therapy, critically ill patient, electrolyte imbalance, fluid

## Abstract

**Objective:**

Continuous renal replacement therapy (CRRT) is the standard treatment for critically ill patients with acute kidney injury (AKI). Electrolyte disturbance such as hypokalemia or hypophosphatemia occurs paradoxically in patients undergoing CRRT due to high clearance. We developed a fluid management protocol for dialysate and replacement fluid that depends on serum electrolytes and focuses on potassium and phosphate levels to prevent electrolyte disturbance during CRRT. The impact of our new fluid protocol on electrolyte stability was evaluated.

**Methods:**

Adult patients who received CRRT between 2013 and 2017 were included. Patients treated 2 years before (2013–2014; pre-protocol group) and 2 years following development of the fluid protocol (2016–2017; protocol group) were compared. The primary outcomes were individual coefficient of variation (CV) and abnormal event rates of serum phosphate and potassium. Secondary outcomes were frequency of electrolyte replacement and incidence of cardiac arrhythmias. Individual CV and abnormal event rates for each electrolyte were analyzed using the Wilcoxon rank-sum test and Chi-square test with Yates’ continuity correction.

**Results:**

A total of 1,448 patients was included. Both serum phosphate and potassium were higher in the protocol group. The CVs of serum phosphate (pre-protocol vs. protocol, 0.275 [0.207–0.358] vs. 0.229 [0.169–0.304], *p* < 0.01) and potassium (0.104 [0.081–0.135] vs. 0.085 [0.064–0.110], *p* < 0.01) were significantly lower in the protocol group. The abnormal event rates of serum phosphate (rate [95% CI], 0.410 [0.400–0.415] vs. 0.280 [0.273–0.286], *p* < 0.01) and potassium (0.205 [0.199–0.211] vs. 0.083 [0.079–0.087], *p* < 0.01) were also significantly lower in the protocol group.

**Conclusion:**

The protocolized management of fluid in CRRT effectively prevented hypophosphatemia and hypokalemia by inducing excellent stability of serum phosphate and potassium levels.

## Introduction

Continuous renal replacement therapy (CRRT) is used widely in critically ill patients with hemodynamic instability (1–3). CRRT is known to induce better hemodynamic stability, fluid balance, and removal of solutes and cytokines, and subsequently to facilitate renal recovery (1, 4–6). Because of the greater clearance and incessant nature of CRRT, overcorrection of electrolytes such as hypophosphatemia and hypokalemia occurs frequently (7, 8). Hypophosphatemia and hypokalemia are common electrolyte disturbances in patients receiving CRRT, with a reported incidence as high as 65 and 24%, respectively (7, 9). Hypophosphatemia can precipitate respiratory muscle dysfunction, weaning failure from mechanical ventilation, myocardial dysfunction, arrhythmia, and rhabdomyolysis (10). Hypokalemia can promote cardiac arrhythmia and muscle weakness (11).

To prevent electrolyte disturbance in critically ill patients receiving CRRT, management of dialysate and replacement solutions should be individualized and reassessed frequently according to serial changes in electrolytes and the condition of the patient (12). CRRT solutions containing different concentrations of potassium and phosphate are available commercially (12). Phoxilium containing 1.2 mmol/L of phosphate and 4.0 mmol/L of potassium can be used for patients at risk of hypophosphatemia and hypokalemia (13). Previous studies reported that Phoxilium increased serum phosphate level and effectively prevented hypophosphatemia during CRRT compared with Hemosol B0 and reduced magnesium supplementation (13, 14). Hypokalemia can be treated with intravenous potassium supplementation and use of CRRT fluid containing 4.0 mmol/L of potassium (15). An algorithm was proposed to determine the appropriate addition of potassium chloride in CRRT fluid to prevent hypokalemia and subsequent arrhythmia (16).

Despite the efforts to prevent electrolyte disturbance during CRRT, there is no standardized protocol for choosing dialysate and replacement fluids. Various studies have been conducted to improve the quality of CRRT operation and to develop standardized guidelines (17, 18). An evidence-based approach and multidisciplinary communication on CRRT application were reported to reduce the duration of CRRT and medical costs (19). We have developed a CRRT fluid protocol prescribing dialysate and replacement fluids according to levels of serum electrolytes focusing on phosphate and potassium. Before applying the protocol with the commercialized solutions containing potassium and phosphate, hypophosphatemia and hypokalemia were managed with by mixing the deficient electrolytes into the CRRT solution or administering them *via* separate fluid. Inevitable time delay for adjusting the composition of CRRT solution or fluid caused hypophosphatemia and hypokalemia with incidence of 29.2 and 18.0%, respectively. This protocol was developed to reduce these electrolytes disturbance and standardize CRRT operation. In this study, we aimed to evaluate the effects of our new CRRT fluid protocol on electrolyte stability and the incidence of electrolyte disturbances.

## Materials and methods

### Study design and patient selection

A total of 1,940 adult patients (≥ 18 years) who received CRRT at Samsung Medical Center from 2013 to 2017 was screened. A fluid protocol for CRRT was developed in 2015, which was defined as the window period. The patients who received CRRT in the pre-protocol period (2013–2014) and the protocol period (2016–2017) were compared. The patients who received CRRT for < 3 days (*n* = 492) or in 2015 were excluded. One cycle of CRRT was defined as the session of CRRT without interruption ≥ 24 h. If CRRT was restarted after 24 h from the termination of CRRT in a same patient, each cycle was regarded as a different case. This study was approved by the Institutional Review Board of Samsung Medical Center in compliance with the Declaration of Helsinki (number: 2020-12-172).

### Fluid protocol for continuous renal replacement therapy

Hemosol B0 (Baxter, Deerfield, IL, United States), MultiBic 4K (Fresenius Medical Care, Bad Homburg, Germany), or Phoxilium (Baxter) were used as dialysate and replacement fluid. The compositions of each fluid are shown in Supplementary Table 1. The primary mode of CRRT was continuous veno-venous hemodiafiltration (CVVHDF), and the initial prescribed dose was 35 mL/kg/h. Approximately 20∼25% of total replacement fluid volume was used as post-replacement fluid. Electrolyte and acid-base status were checked every 6–8 h depending on the condition of the patient. Blood was collected from patient’s peripheral veins or central venous line that is not used for drug or fluid infusion. In the protocol, dialysate and pre-replacement fluid were adjusted every 6–8 h according to serial changes in serum potassium and phosphate (Table 1). Phoxilium was used as both dialysate and pre-replacement fluid if serum phosphate and potassium levels were ≤ 3.5 and ≤ 4.5 mmol/L, respectively. MultiBic 4K was used as dialysate until serum potassium level reached 5.1 mmol/L if serum phosphate level was ≥ 3.6 mg/dL. Hemosol B0 was used as post-replacement fluid. In a study of dialysis patients, all-cause mortality and cardiovascular mortality were low when predialysis potassium was between 4.6 and 5.6 mmol/L (20). In another study, the use of low potassium dialysate (potassium less than 2 mmol/L) increased probability of sudden cardiac arrest when serum potassium was below 5.0 mmol/L (21). Almost 50% of CRRT patients had heart disease in our cohort, in which the potassium target was required to be set as higher than median value of normal range by cardiologists and thoracic surgeons. Therefore, the reference value of potassium was determined as 4.5 mmol/L (0.2 mmol/L higher than median value) and 5.1 mmol/L (upper limit of normal range), respectively. In dialysis patients, serum phosphate levels showed U-shaped association in all-cause mortality (22). The normal range of serum phosphate levels in our center is 2.5–4.5 mg/dL, therefore the reference value of phosphate was determined as 3.5 mg/dL which is the median. In patients with persistent or progressive hypercalcemia (ionized calcium ≥ 1.45 mmol/L) and underlying disease-causing hypercalcemia, Hemosol B0 without reconstitution of the A compartment containing calcium was used as pre- and post-replacement fluids (Supplementary Table 2). Structured order set including the contents of this CRRT fluid protocol was created and incorporated into EMR. Especially, CRRT fluid protocol was inserted as a table of a convenient pop-up window in an order communication system for nurses, so that nurses could conduct changing CRRT solutions according to the protocol immediately when the laboratory results were reported.

**TABLE 1:** CRRT fluid protocol according to serum phosphate and potassium levels.

	Dialysate		Pre-replacement fluid		Post-replacement fluid
			
Serum level	*P* ≥ 3.6	*P* ≤ 3.5	*P* ≥ 3.6	*P* ≤ 3.5	
K ≤ 4.5	MultiBic 4K	Phoxilium	MultiBic 4K	Phoxilium	
4.6 ≤ K ≤ 5.0	MultiBic 4K	Phoxilium	Hemosol B0	Hemosol B0	Hemosol B0
K ≥ 5.1	Hemosol B0	Hemosol B0	Hemosol B0	Hemosol B0	

Severe chronic hyponatremia: Hyponatremia (<125 mmol/L) lasting more than 48 h. Patient who had hyponatremia at the time of admission was defined as chronic hyponatremia.

1) Administration of dextrose water was initiated at a rate of 1.5 mL/kg/h at the beginning of CRRT.

2) Electrolyte tests: followed at 4-h intervals.

3) Adjust the infusion rate of dextrose water targeting ΔNa ≤ 2 mmol/L for 4 h.

4) Maintain dextrose water if ΔNa is 1 mmol/L for 4 h.

5) Reduce the infusion rate of dextrose water if ΔNa < 1 mmol/L for 4 h.

6) If ΔNa > 2 mmol/L for 4 h, increase the infusion rate of dextrose water to 2–2.5 mL/kg/h.

7) When hyponatremia was reliably and slowly corrected by 24 h after the initiation of CRRT, checking intervals of electrolytes were adjusted to every 6 h.

Criteria for electrolyte supplementation.

1) P < 1.5 mg/dL or symptoms were present: potassium phosphate IV.

2) K < 3.0 mmol/L: potassium chloride IV.

3) Mg < 1.5 mg/dL: magnesium sulfate IV.

4) iCa < 0.9 mmol/L or total serum Ca < 7.5 mg/dL or acute symptoms were present: calcium gluconate IV.

5) 1.5 ≤ P ≤ 2.4, 3.0 ≤ K ≤ 3.4, 1.5 ≤ Mg ≤ 1.8, 0.9 ≤ iCa ≤ 1.04: consider supplementations orally.

Ca, calcium; CRRT, continuous renal replacement therapy; iCa, ionized calcium (mmol/L); IV, intravenously; K, potassium (mmol/L); Mg, magnesium; P, phosphate (mg/dL); ΔNa, changes in serum sodium concentration.

Dextrose water with an initial infusion rate of 1.5 mL/kg/h was administered to patients with preexisting severe chronic hyponatremia to prevent excessively rapid correction of hyponatremia. Chronic hyponatremia was defined as hyponatremia lasting > 48 h. Hyponatremia diagnosed at the time of admission was regarded as chronic hyponatremia. When serum sodium concentration was < 125 mmol/L, a separate administration of dextrose water was initiated at the same time starting CRRT. All administered volume of separately infused fluids including dextrose water was included in the input volume for calculating volume removal through CRRT, therefore additional volume by dextrose water infusion was adequately removed according to volume management order usually prescribed in CRRT. Sodium chloride or sodium bicarbonate was appropriately mixed into CRRT fluid for maintaining hypernatremia in patients with cerebral edema and for preventing a too rapid correction of severe hypernatremia. Severe hypernatremia was defined as serum sodium concentration ≥ 155 mmol/L. Sodium chloride was selected when patients had only hypernatremia without significant metabolic acidosis. Sodium bicarbonate was selected for patients with hypernatremia and significant metabolic acidosis with serum bicarbonate < 15 mmol/L. Sodium chloride or sodium bicarbonate mixed in the dialysate and replacement fluid to sodium concentration of about 5 mmol/L lower than serum sodium level. In such cases, the first follow-up of electrolytes was performed at 2 h after applying the protocol. If the sodium level was stable, the follow-up interval was increased to 4 h. Severe metabolic acidosis (pH < 7.2 or HCO_3_ < 10 mmol/L) was managed in three steps as follows; infusion of bicarbonate fluid by a separate line, increment of CRRT dose, and addition of sodium bicarbonate in dialysate and replacement fluids up to 70 mmol in a 5 L-solution bag. The degree of bicarbonate deficit was calculated and mixed up to 7 ample of bicarbonate in 1 L of dextrose water. The CRRT dose was increased to maximum 65–85 mL/kg/h. When metabolic acidosis improved significantly, the appliance of these methods was stopped in inverse order; addition of sodium bicarbonate into CRRT fluids was stopped, the dose of CRRT was decreased, and bicarbonate fluid infusion was reduced or stopped. Intravenous or oral supplementation of electrolytes was appropriately prescribed as needed (Table 1).

A total of 17 CRRT machines was used to treat patients in 128 ICU beds cared by a multidisciplinary team. Our center operated dedicated nursing team for CRRT to minimize the technical problems and interrupting time of CRRT. CRRT was performed using Prismaflex (Baxter) with ST100 filter. If filters were maintained for ≥ 24 h, no anticoagulants were used. If the maintenance time of filters was < 24 h, the use of anticoagulants was considered. Nafamostat mesylate was used when platelets < 100,000/μL, activated partial thromboplastin time (aPTT) > 60 s, or prothrombin time-international normalized ratio (PT INR) > 2.0. Nafamostat was also used in patients with hemorrhagic lesions or major surgery within 2 weeks, cerebral hemorrhage within the last 3 months, or disseminated intravascular coagulation. Administration of nafamostat was started at a rate of 10 mg/h and the dose was increased to 20 mg/h, as necessary. When the risk of bleeding was low, heparin was used with Initial bolus administration of 1,000–2,000 IU followed by continuous intravenous infusion at a rate of 5–10 IU/kg/h.

### Clinical and laboratory data collection

Clinical data of age, sex, comorbidities, mean arterial pressure (MAP), body weight, and duration of CRRT were collected from electronic medical records (EMR). Laboratory data of blood urea nitrogen (BUN), serum creatinine, serum phosphate, potassium, sodium, magnesium, ionized calcium, and bicarbonate were also collected. All measured values of each electrolyte were collected throughout the whole duration of CRRT. The medical cost of the CRRT operation was also collected.

### Outcomes

To evaluate the stability of electrolytes, the coefficient of variation (CV) and abnormal event rates of each electrolyte were defined as the primary outcomes. Abnormal event rates were defined as ratios of the measurements showing abnormal ranges in all measurements. Subgroup analysis was performed by dividing the abnormal event rate into hypo-abnormal events and hyper-abnormal events. Each abnormal event was defined as follows: phosphate (hypophosphatemia≤2.4 mg/dL, hyperphosphatemia≥4.6 mg/dL), potassium (hypokalemia≤3.4 mmol/L, hyperkalemia≥5.2mmol/L), sodium (hyponatremia ≤135mmol/L, hypernatremia≥146mmol/L), magnesium (hypomagnesemia≤1.8mg/dL, hypermagnesemia ≥2.6mg/dL), and ionized calcium (hypocalcemia≤1.04 mmol/L, hypercalcemia≥1.36 mmol/L). The frequency of supplementation treatment for deficient electrolytes and the incidence of cardiac arrhythmias requiring anti-arrhythmic or rate-controlling medications or defibrillation were defined as the secondary outcomes. In-hospital mortality was also included as a secondary outcome.

### Statistical analysis

Continuous variables are expressed as medians with interquartile ranges (IQRs) and were compared using the Mann-Whitney test. Categorical variables were compared by Chi-square test or Fisher’s exact test. The coefficient of variation (CV) was calculated as the ratio of the standard deviation (SD) to the mean (μ). The CV is one of the most popular indices that estimate the variability in repeatedly measured values (e.g., day-to-day blood pressure levels, visit-to-visit glucose levels) during a certain period (23–25). The SD and variability independent of the mean (VIM) are also commonly used to estimate variability. The CV has some advantages in the independency of the average of those repeatedly measured values against the SD and in the straightforwardness of calculation against the VIM. The abnormal event rates of each electrolyte were calculated as the ratios of the number of abnormal measurements to the total number of measurements. CV and the abnormal event rates were calculated for serum phosphate, potassium, sodium, magnesium, ionized calcium, and bicarbonate. Wilcoxon rank-sum test was used to compare CV and the abnormal event rates between the pre-protocol group and the protocol group. The abnormal event rates of each electrolyte and 95% confidence interval (CI) were also calculated as the fraction of the total. Chi-square test with Yates’ continuity correction was used to compare the abnormal event rates between the two groups. When calculating CV and abnormal event rates, baseline values right before CRRT were excluded considering the purpose of this study. Cases with fewer than 5 total measurements during CRRT were excluded from the final analyses. To adjust for confounding factors due to CRRT interruption, cases in which the CV value exceed sum of mean and SD times 3 were defined as an outlier, and all outliers were thoroughly reviewed through EMR. The patients, in whom CRRT was interrupted for > 1 hour, were excluded in the final analysis. Although separate fluid therapy could not be adjusted, the administration of fluid was performed according to the ICU protocol in both groups. CV and abnormal event rates were analyzed by dividing into two subgroups according to whether the baseline measurement values were in normal or abnormal range.

Total medical costs of CRRT were calculated by multiplying the estimated cost per unit and total duration of CRRT. The length of CRRT and CRRT-related costs of the groups were compared using the rank-sum test and linear mixed models for longitudinal data with random intercepts. In the mixed model, log-transformed data of log (values + 1) were used for the primary outcomes. The ratios with 95% CIs were used to compare the length and costs of CRRT because the data were markedly right-skewed.

A *p*-value < 0.05 was considered statistically significant. All statistical analyses were conducted using IBM SPSS Statistics 25 (IBM Corporation, Armonk, NY, United States), SAS version 9.4 (SAS Institute Inc., Cary, NC, United States), or STATA version 16 (StataCorp LP, College Station, TX, United States).

## Results

### Study flow and patient characteristics

**FIGURE 1:**
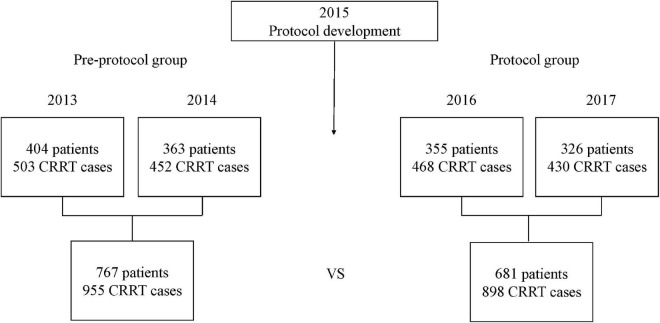
Study design. The CRRT fluid protocol was developed in 2015. Of the patients who received CRRT for more than 3 days, 767 were in the pre-protocol period (2013–2014) and 681 were in the protocol period (2016–2017). A total of 955 cases in the pre-protocol group and 898 cases in the protocol group were compared. CRRT, continuous renal replacement therapy.

In the patients who received CRRT for more than 3 days, 767 were in the pre-protocol period (2013–2014) and 681 were in the protocol period (2016–2017). Totals of 955 and 898 cases were compared in each period, respectively (Figure 1). Baseline characteristics of all patients are shown in Table 2. The median age was 63 years, and the median treatment duration was 4 days. Sex ratio and comorbidities such as diabetes mellitus, hypertension, and chronic kidney disease were comparable in the two groups. Baseline serum phosphate and potassium were similar between the two groups. Patients in the protocol group had higher baseline sodium, magnesium, bicarbonate, and lower ionized calcium than the pre-protocol group.

**TABLE 2:** Baseline characteristics.

	Total (*n* = 1,853)	Pre-protocol (*n* = 955)	Protocol (*n* = 898)	*P*-value
Age, year	63.0 [54.0–72.0]	64.0 [54.0–72.0]	63.0 [53.0–72.0]	0.46
Male, *n* (%)	1,194 (64.2%)	618 (64.3%)	572 (63.6%)	0.38
CRRT duration, day	4.0 [3.0–8.0]	4.0 [30–7.0]	4.0 [0.20–0.60]	0.11
Body weight, kg	62.9 [54.9–71.7]	61.9 [53.8–71.0]	63.9 [56.0–72.4]	<0.01
MAP, mmHg	72.0 [64.0–84.0]	73.0 [65.0–84.0]	71.0 [63.0–83.0]	<0.01
** Comorbid condition, n (%)**				
Diabetes mellitus	730 (39.2%)	372 (38.7%)	385 (39.8%)	0.64
Hypertension	959 (51.5%)	479 (49.8%)	480 (53.3%)	0.13
Ischemic heart disease	220 (11.8%)	97 (10.1%)	123 (13.7%)	0.02
Heart failure	366 (19.7%)	162 (16.9%)	204 (22.7%)	<0.01
Cerebrovascular disease	11 (0.6%)	5 (0.5%)	6 (0.7%)	0.68
Chronic kidney disease	606 (32.6%)	299 (31.1%)	307 (34.1%)	0.17
** Laboratory variables at initiation of CRRT**				
BUN, mg/dL	55.7 [35.2–79.8]	52.8 [33.0–77.1]	57.7 [38.2–83.5]	<0.01
Serum creatinine, mg/dL	2.79 [1.77–4.40]	2.76 [1.72–4.47]	2.81 [1.81–4.25]	0.37
P, mg/Dl	4.5 [3.4–5.8]	4.5 [3.4–5.8]	4.5 [3.4–5.8]	0.31
K, mmol/L	4.4 [3.9–5.1]	4.3 [3.8–5.0]	4.5 [4.0–5.1]	0.84
Na, mmol/L	136 [132–141]	136 [132–140]	137 [133–141]	<0.01
Mg, mg/dL	2.2 [2.0–2.5]	2.1 [1.9–2.4]	2.3 [2.1–2.7]	<0.01
iCa, mmol/L	1.11 [1.04–1.19]	1.12 [1.04–1.21]	1.10 [1.04–1.18]	<0.01
HCO3, mmol/L	19.3 [15.4–23.0]	18.9 [14.7–23.0]	19.6 [16.2–23.0]	<0.01

BUN, blood urea nitrogen; CRRT, continuous renal replacement therapy; HCO3, bicarbonate; iCa, ionized calcium; K, potassium; MAP, mean arterial pressure; Mg, magnesium; Na, sodium; P, phosphate.

### Electrolyte levels during continuous renal replacement therapy

The median levels of each electrolyte during CRRT are shown in Supplementary Table 3. Serum phosphate (pre-protocol vs. protocol groups, median [IQR], mg/dL, 3.0 [2.4–3.8] vs. 3.2 [2.7–3.9], *p* < 0.01) and potassium (mmol/L, 3.8 [3.6–4.2] vs. 4.2 [3.9–4.5], *p* < 0.01) during CRRT were higher in the protocol group. Serum sodium (mmol/L, 135 [133–137] vs. 137 [135–139], *p* < 0.01) and magnesium (mg/dL, 2.0 [1.9–2.2] vs. 2.1 [1.9–2.3], *p* < 0.01) during CRRT were also higher in the protocol group. Serum ionized calcium was lower in the protocol group (mmol/L, 1.24 [1.16–1.3] vs. 1.16 [1.11–1.22], *p* < 0.01).

### Variability of electrolytes evaluated with coefficient of variations

**FIGURE 2:**
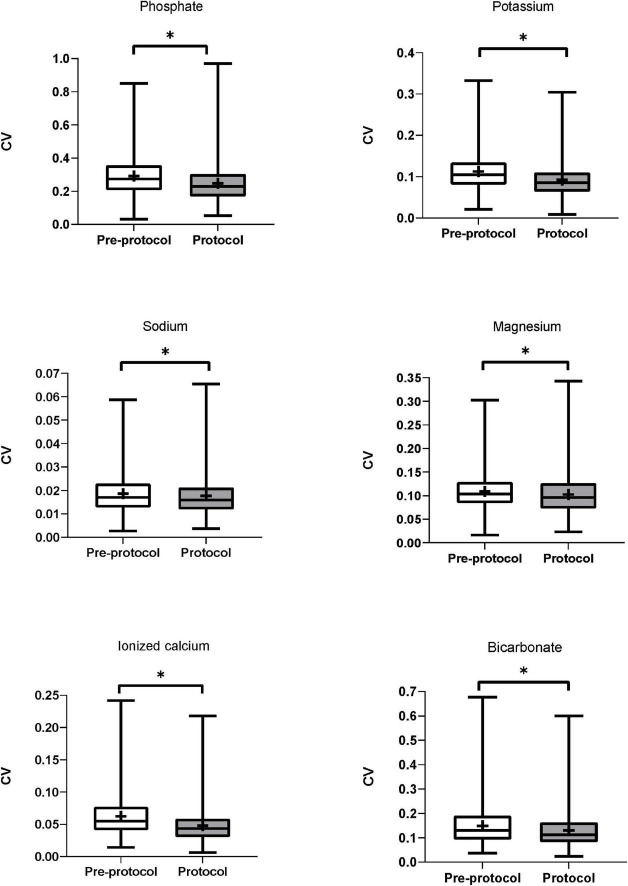
Coefficient of variation of serum electrolytes. CVs of serum phosphate, potassium, sodium, magnesium, ionized calcium, and bicarbonate were significantly lower in the protocol group than in the pre-protocol group. CV, coefficient of variation. * Means *p*-value < 0.05.

The CVs of serum phosphate (pre-protocol vs. protocol groups, median [IQR], 0.274 [0.207–0.357] vs. 0.229 [0.169–0.304], *p* < 0.01) and serum potassium (0.104 [0.081–0.134] vs. 0.085 [0.064–0.110], *p* < 0.01) were significantly lower in the protocol group (Table 3). Subgroup analysis of serum phosphate and potassium according to normality of baseline values showed lower CVs in the protocol group (Supplementary Table 4). The CVs of serum sodium (0.017 [0.013–0.023] vs. 0.016 [0.012–0.021], *p* < 0.01), magnesium (0.103 [0.084–0.129] vs. 0.096 [0.073–0.126], *p* < 0.01), ionized calcium (0.055 [0.041–0.077] vs. 0.043 [0.031–0.059], *p* < 0.01), and bicarbonate (0.131 [0.093–0.190] vs. 0.112 [0.083–0.163], *p* < 0.01) were also significantly lower in the protocol group (Figure 2). Subgroup analysis of each electrolyte according to normality of baseline values showed lower CVs in the protocol group except in cases of abnormal baseline Na and normal baseline bicarbonate (Supplementary Table 4).

**TABLE 3:** Coefficient of variation of each electrolyte.

Serum electrolytes	Group	Number of cases	CV, median [IQR]	*P*-value
P	Total	1,694	0.251 [0.186–0.333]	–
	Pre-protocol	817	0.274 [0.207–0.357]	<0.01
	Protocol	877	0.229 [0.169–0.304]	
K	Total	1764	0.094 [0.072–0.122]	–
	Pre-protocol	875	0.104 [0.081–0.134]	<0.01
	Protocol	889	0.085 [0.064–0.110]	
Na	Total	1761	0.016 [0.012–0.022]	–
	Pre-protocol	873	0.017 [0.013–0.023]	<0.01
	Protocol	888	0.016 [0.012–0.021]	
Mg	Total	1547	0.100 [0.077–0.128]	–
	Pre-protocol	680	0.103 [0.084–0.129]	<0.01
	Protocol	867	0.096 [0.073–0.126]	
iCa	Total	1620	0.049 [0.035–0.049]	–
	Pre-protocol	758	0.055 [0.041–0.077]	<0.01
	Protocol	862	0.043 [0.031–0.059]	
HCO3	Total	1791	0.121 [0.087–0.176]	–
	Pre-protocol	921	0.131 [0.093–0.190]	<0.01
	Protocol	870	0.112 [0.083–0.163]	

CV, coefficient of variation; HCO3, bicarbonate; iCa, ionized calcium; K, potassium; Mg, magnesium; Na, sodium; P, phosphate.

### Variability of electrolytes evaluated with abnormal event rates

**FIGURE 3:**
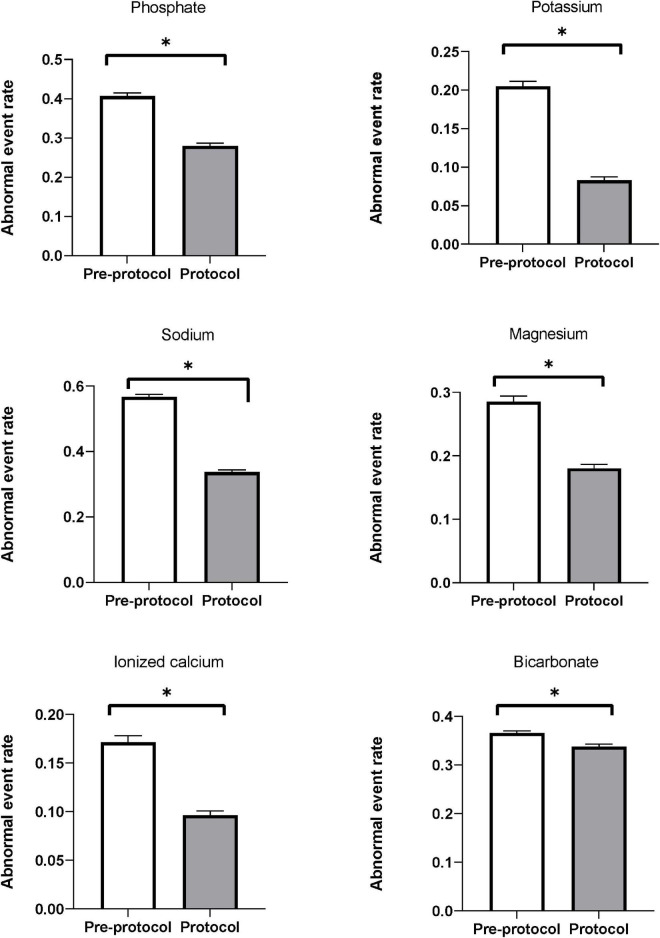
Abnormal event rates of serum electrolytes. Abnormal event rates (the ratio of measurements showing abnormal ranges to all measurements) of serum phosphate, potassium, sodium, magnesium, ionized calcium, and bicarbonate were significantly decreased after applying the protocol. * Means *p*-value < 0.05.

Figure 3 shows the abnormal event rates of each electrolyte. The abnormal event rates of serum phosphate (pre-protocol vs. protocol, rates [95% CI], 0.410 [0.398–0.415] vs. 0.280 [0.273–0.286], *p* < 0.01) and potassium (0.205 [0.199–0.211] vs. 0.083 [0.079–0.087], *p* < 0.01) were significantly lower in the protocol group than in the pre-protocol group (Table 4). Subgroup analysis according to normality of baseline serum phosphate and potassium levels also showed significant lower abnormal event rates in the protocol group than in the pre-protocol group (Supplementary Table 5). The abnormal event rates of serum sodium (0.567 [0.559–0.575] vs. 0.337 [0.330–0.344], *p* < 0.01), magnesium (0.285 [0.277–0.294] vs. 0.180 [0.174–0.187], *p* < 0.01), ionized calcium (0.171 [0.165–0.178] vs. 0.096 [0.092–0.101], *p* < 0.01), and bicarbonate (0.366 [0.361–0.370] vs. 0.338 [0.333–0.343], *p* < 0.01) were also significantly lower in the protocol group (Table 4). Hypo-abnormal event rates and hyper-abnormal event rates are shown in the Supplementary Table 6. The hypo-abnormal event rates of phosphate, potassium, sodium, and magnesium were lower in the protocol group. Although hyper-abnormal event rates of potassium, sodium, magnesium were higher in the protocol group, the protocol group did not show clinically significant high electrolyte values considering the distribution of total electrolyte values (Supplementary Table 3).

**TABLE 4:** Abnormal event rates of each electrolyte.

Serum electrolytes	Group	Number of observations in normal range	Number of observations in abnormal range	Total observations	Abnormal event rate	95% CI	*P*-value
P	Total	19,278	9788	29,066	0.337	0.331–0.342	–
	Pre-protocol	7,794	5,334	13,128	0.410	0.398–0.415	<0.01
	Protocol	11,484	4,454	15,938	0.280	0.273–0.286	
K	Total	27,715	4,556	32,271	0.141	0.137–0.145	–
	Pre-protocol	12,261	3,160	15,421	0.205	0.199–0.211	<0.01
	Protocol	27,715	4,556	32,271	0.083	0.079–0.087	
Na	Total	17,822	14,367	32,189	0.446	0.441–0.452	–
	Pre-protocol	6,645	8,692	15,337	0.567	0.559–0.575	<0.01
	Protocol	11,177	5,675	16,852	0.337	0.330–0.344	
Mg	Total	20,386	5,839	26,225	0.223	0.218–0.228	–
	Pre-protocol	7,541	3,011	10,552	0.285	0.277–0.294	<0.01
	Protocol	12,845	2,828	15,673	0.180	0.174–0.187	
iCa	Total	23,982	3,538	27,520	0.129	0.125–0.133	–
	Pre-protocol	9,822	2,030	11,852	0.171	0.165–0.178	<0.01
	Protocol	14,160	1,508	15,668	0.096	0.092–0.101	
HCO3	Total	49,312	26,975	76,287	0.354	0.350–0.357	–
	Pre-protocol	27,210	15,691	42,901	0.366	0.361–0.370	<0.01
	Protocol	22,102	11,284	33,386	0.338	0.333–0.343	

HCO3, bicarbonate; iCa, ionized calcium; K, potassium; Mg, magnesium; Na, sodium; P, phosphate.

Phosphate: normal range (2.5–4.5 mg/dL), abnormal range (≤ 2.4 mg/dL, ≥ 4.6 mg/dL).

Potassium: normal range (3.5–5.1 mmol/L), abnormal range (≤ 3.4 mmol/L, ≥ 5.2 mmol/L).

Sodium: normal range (136–145 mmol/L), abnormal range (≤ 135 mmol/L, ≥ 146 mmol/L).

Magnesium: normal range (1.9–2.5 mg/dL), abnormal range (≤ 1.8 mg/dL, ≥ 2.6 mg/dL).

Ionized calcium: normal range (1.05–1.35 mmol/L), abnormal range (≤ 1.04 mmol/L, ≥ 1.36 mmol/L).

Bicarbonate: normal range (≥ 20 mg/dL), abnormal range (< 20 mg/dL).

### Secondary outcomes

**FIGURE 4:**
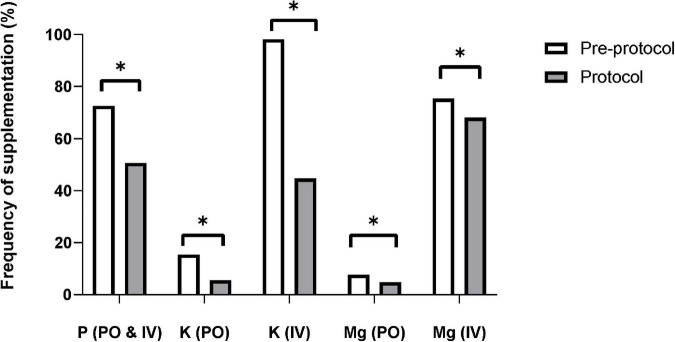
Frequency of electrolyte supplementation. The supplementation frequencies of phosphate, potassium, and magnesium were significantly decreased after applying the protocol. IV, intravenous; K, potassium; Mg, magnesium; P, phosphate; PO, per oral. * Means *p*-value < 0.05.

The frequencies of phosphate, potassium, and magnesium supplementation were significantly lower in the protocol group than in the pre-protocol group (Figure 4). The incidence of cardiac arrhythmia requiring anti-arrhythmic or rate-controlling medication was similar in the two groups [pre-protocol vs. protocol, *n* (%), 141(14.7%) vs. 139 (15.4%), *p* = 0.64], as was the frequency of defibrillation [57 (5.9%) vs. 64 (7.1%), *p* = 0.30]. In-hospital mortality rate was lower in protocol group [515 (53.9%) vs. 417 (46.3%), *p* = 0.001].

### Cost analysis

The median length of CRRT was 4 days. The duration of CRRT was shorter in the protocol group than in the pre-protocol group (ratio = 0.94, 95% CI = 0.89–0.99, *p* = 0.04). The median cost related to CRRT was 2,012 USD and 1,884 USD in the pre-protocol and protocol groups, respectively. Total cost was lower by an average of 6% in the protocol group than in the pre-protocol group (ratio = 0.94, 95% CI = 0.89–0.99, *p* = 0.045). The cost of electrolyte supplementation was much lower in the protocol group than in the pre-protocol group (ratio = 0.21, 95% CI = 0.17–0.27, *p* < 0.01) (Supplementary Table 7).

## Discussion

In this study, we analyzed the effects of protocolized fluid management on the stability of electrolytes in patients receiving CRRT. Serum phosphate, potassium, sodium, magnesium, ionized calcium, and bicarbonate were significantly more stable in the protocol group. The abnormal event rates associated with serum phosphate, potassium, sodium, magnesium, ionized calcium, and bicarbonate were also significantly lower in the protocol group. Our fluid management protocol significantly reduced electrolyte variability and imbalance during CRRT.

Hypophosphatemia is associated with ventilator weaning failure related to respiratory muscle dysfunction (26), myocardial dysfunction and arrhythmia (27), and increased mortality (28). Hypophosphatemia during CRRT can be prevented by use of phosphate supplementation when initiating CRRT (29) and using phosphate-containing CRRT solutions (13, 14, 30). Phoxilium, CRRT solution containing phosphate of 1.2 mmol/L, was found to reduce the episodes of hypophosphatemia during CRRT in several studies (13, 14, 31). Consistent with previous studies, the mean serum phosphate was significantly increased after the protocol was applied in our study. Both the variation and abnormal event rates of serum phosphate were significantly decreased by our protocol. In particular, the abnormal event rates decreased by 31.2% after applying the protocol.

Hypokalemia can promote or aggravate cardiac arrhythmia (11). A rapid electrolyte shift, especially that of potassium, can increase sensitivity to cardiac arrhythmia (32, 33) and can trigger sudden cardiac death in hemodialysis patients (34). Previous studies have reported that low potassium dialysate was associated with high risk of sudden cardiac arrest in hemodialysis patients (21, 35). To prevent hypokalemia during CRRT, one study used electrolyte-enriched CRRT solution and reduced hypokalemia (36). In our protocol, we also used CRRT fluids containing 4 mmol/L potassium, and the mean serum potassium level was higher in the protocol group than in the pre-protocol group. Furthermore, our protocol reduced the variability and abnormal event rates of serum potassium during CRRT by 59.6%.

Hyponatremia and hypernatremia occur in approximately 30 and 15% of ICU patients, respectively (37, 38). The correction rate of chronic dysnatremia is crucial because rapid correction can lead to osmotic demyelination syndrome or brain edema (39). The correction rate of serum sodium should not exceed 10 mmol/L over 24 h in patients with chronic dysnatremia (39, 40). Rapid correction of serum sodium level is inevitable with CRRT solutions containing 140 mmol/L of sodium (40). The addition of free water and step-wise adjustment of sodium level in CRRT solutions were reported to prevent rapid correction of hyponatremia safely and effectively (15, 40). Similarly, rapid correction of chronic hypernatremia could be prevented by addition of 30% NaCl (40, 41). Administration of 5% dextrose water or 3% saline with a separate line or a return limb in the CRRT circuit was reported to attenuate rapid changes in serum sodium level (15). In our study, the variability of serum sodium levels was significantly decreased by the protocol. In addition, our study supports the separate administration of dextrose fluid for correcting hyponatremia more stably with less risk of rapid correction during CRRT in patients with chronic hyponatremia.

Hypocalcemia is also common in critically ill patients (42) and can be precipitated by citrate anticoagulation during CRRT (43, 44). Calcium is a crucial factor for maintaining cellular function and triggering muscle contraction (45, 46). Hypocalcemia is associated with higher mortality in critically ill patients (47). Hypocalcemia tends to be more frequent during CRRT than during intermittent hemodialysis because dialysates containing a relatively higher concentration of calcium are usually used for intermittent hemodialysis (48). In our protocol, we used Hemosol B0 without calcium as a replacement fluid in patients with progressive or persistent hypercalcemia and underlying disease that caused hypercalcemia. The variation of serum ionized calcium was significantly decreased by the protocol. In particular, the abnormal event rate of serum calcium was decreased by 43.8% in the protocol group. However, these results might be different from those of centers using regional citrate anticoagulation, since nafamostat or heparin were used as anticoagulants in our center.

Magnesium can be lost through effluents during CRRT (49). In a study investigating mineral loss during CRRT, mean serum magnesium was significantly lower in the CRRT group than in the control group not treated with CRRT (49). CRRT fluids containing 0.7 mmol/L of magnesium were reported to maintain serum magnesium level in a normal range (50). In a previous study, the use of Phoxilium as dialysis fluid reduced the frequency of magnesium supplementation (14). In our protocol, CRRT fluids containing 0.5–0.6 mmol/L of magnesium were used, and the median serum level of magnesium was maintained in a normal range with less variation, and the frequency of magnesium supplementation decreased significantly after applying the protocol.

Commercial CRRT fluids contain up to 35 mmol/L bicarbonate as a buffer. Most acute kidney injury (AKI) patients who require CRRT have severe metabolic acidosis; therefore, metabolic acidosis is a common primary indication of CRRT (15). In our protocol, sodium bicarbonate was added to the CRRT fluids of patients with severe metabolic acidosis that was not easily corrected by commercial CRRT fluids. However, paradoxical metabolic alkalosis might occur due to overcorrection of metabolic acidosis during CRRT (15). In our protocol, through frequent monitoring of acid-base status in patients and appropriate adjustment of CRRT dose and bicarbonate in CRRT fluids, the serum bicarbonate level was maintained stably with a median level of 21.4 mmol/L and less variation.

The previous studies that aimed to prevent hypophosphatemia and hypokalemia during CRRT compared Phoxilium with other CRRT fluids not containing phosphate or potassium (13, 14, 36). These studies monitored serum electrolytes regularly but did not change the type of CRRT fluid according to serum potassium or phosphate level. In contrast, our study monitored serum electrolytes every 6–8 h and changed the type of CRRT fluid immediately according to the level of serum potassium or phosphate. Our protocol fixed Hemosol B0 as post-replacement fluid to prevent unintended hyperphosphatemia and hyperkalemia. Under the protocol, the ICU nursing team could change CRRT fluids easily and promptly without time delay for reporting to a physician. Additional manipulation of CRRT fluids through mixing phosphate or potassium was avoided by the protocol to minimize contamination risk. Our protocol showed significant stability not only for phosphate and potassium, but also sodium, magnesium, ionized calcium, and bicarbonate. Regardless of the baseline values, the variations of serum phosphate and potassium were significantly decreased by the protocol. The supplementation frequencies of phosphate, potassium, and magnesium were decreased significantly after applying the protocol. These results can contribute to reducing workload and medical cost and handling the shortage of supplementation medications (51).

In our study, the total cost associated with CRRT was lower by an average of 6% in the protocol group than in the pre-protocol group. The cost of CRRT fluid was not different between the two groups. However, management costs and electrolyte supplementation costs were significantly reduced in the protocol group. AKI has been recognized as a global medical burden (52). Length of hospital stay and costs were known to increase proportionally with the severity of AKI (53). The cost benefits we demonstrated suggest that an appropriate management of the CRRT fluid protocol is an alternative strategy for reducing medical costs in patients with severe AKI.

Our study has several limitations. First, potential bias caused by a retrospective design might confound the study results. To overcome this limitation, we analyzed a sufficient number of measurements based on a large cohort. Second, a hard outcome such as mortality was not included in our study. We compared the administration frequency of anti-arrhythmic or rate-controlling medication and defibrillation, but there were no significant differences between the two groups. It seemed that cardiac arrhythmia depends not only on electrolyte disturbance, but also on underlying heart disease. Third, we did not analyze the long-term renal outcome because the main purpose of this study was to investigate the clinical relevance of protocolized fluid management focusing on electrolyte stability during CRRT.

In conclusion, protocolized fluid management improved the stability of electrolytes, especially phosphate and potassium, during CRRT. The CVs and abnormal event rates of serum phosphate, potassium, sodium, magnesium, ionized calcium, and bicarbonate were significantly decreased by the protocol. Protocolized CRRT fluid management resulted in excellent stability of serum electrolyte levels. Further prospective studies are required to evaluate the long-term effects of our novel CRRT fluid protocol.

## Data availability statement

The raw data supporting the conclusions of this article will be made available by the authors, without undue reservation.

## Ethics statement

The studies involving human participants were reviewed and approved by the Institutional Review Board of Samsung Medical Center. The ethics committee waived the requirement of written informed consent for participation.

## Author contributions

SB and HJ: research idea, study design, data analysis and interpretation, and writing—original draft. SB: data acquisition. SB, DK, Y-GK, DJK, and KK: statistical analysis. HJ: supervision. JJ, SN, JY, CC, JL, WH, and GS: writing—review and editing. All authors contributed important intellectual content during manuscript drafting or revision, accepts personal accountability for their contributions, and agrees to ensure that questions pertaining to the accuracy or integrity of any portion of the work are appropriately investigated and resolved.

## References

[1] Vanholder R Van Biesen W Lameire N. What is the renal replacement method of first choice for intensive care patients? *J Am Soc Nephrol.* (2001) 12:S40–3. 10.1681/ASN.V12suppl_1s40 11251030

[2] Jiang L Zhu Y Luo X Wen Y Du B Wang M Epidemiology of acute kidney injury in intensive care units in Beijing: The multi-center BAKIT study. *BMC Nephrol.* (2019) 20:468. 10.1186/s12882-019-1660-z 31842787 PMC6915890

[3] Park S Lee S Jo HA Han K Kim Y An JN Epidemiology of continuous renal replacement therapy in Korea: Results from the national health insurance service claims database from 2005 to 2016. *Kidney Res Clin Pract.* (2018) 37:119–29. 10.23876/j.krcp.2018.37.2.119 29971207 PMC6027810

[4] Augustine JJ Sandy D Seifert TH Paganini EP. A randomized controlled trial comparing intermittent with continuous dialysis in patients with ARF. *Am J Kidney Dis.* (2004) 44:1000–7. 10.1053/j.ajkd.2004.08.022 15558520

[5] De Vriese AS Colardyn FA Philippe JJ Vanholder RC De Sutter JH Lameire NH. Cytokine removal during continuous hemofiltration in septic patients. *J Am Soc Nephrol.* (1999) 10:846–53. 10.1681/ASN.V104846 10203370

[6] Bonnassieux M Duclos A Schneider AG Schmidt A Benard S Cancalon C Renal replacement therapy modality in the ICU and renal recovery at hospital discharge. *Crit Care Med.* (2018) 46:e102–10. 10.1097/CCM.0000000000002796 29088005

[7] Bellomo R Cass A Cole L Finfer S Gallagher M Lo S Intensity of continuous renal-replacement therapy in critically ill patients. *N Engl J Med.* (2009) 361:1627–38. 10.1056/NEJMoa0902413 19846848

[8] Jung SY Kim H Park S Jhee JH Yun HR Kim H Electrolyte and mineral disturbances in septic acute kidney injury patients undergoing continuous renal replacement therapy. *Medicine (Baltimore).* (2016) 95:e4542. 10.1097/MD.0000000000004542 27603344 PMC5023866

[9] Rhee H Berenger B Mehta RL Macedo E. Regional citrate anticoagulation for continuous kidney replacement therapy with calcium-containing solutions: A cohort study. *Am J Kidney Dis.* (2021) 78:550.e–9.e. 10.1053/j.ajkd.2021.01.017 33798636 PMC8723918

[10] Geerse DA Bindels AJ Kuiper MA Roos AN Spronk PE Schultz MJ. Treatment of hypophosphatemia in the intensive care unit: A review. *Crit Care.* (2010) 14:R147. 10.1186/cc9215 20682049 PMC2945130

[11] Tazmini K Frisk M Lewalle A Laasmaa M Morotti S Lipsett DB Hypokalemia promotes arrhythmia by distinct mechanisms in atrial and ventricular myocytes. *Circ Res.* (2020) 126:889–906. 10.1161/CIRCRESAHA.119.315641 32070187 PMC7098435

[12] Murugan R Hoste E Mehta RL Samoni S Ding X Rosner MH Precision fluid management in continuous renal replacement therapy. *Blood Purif.* (2016) 42:266–78. 10.1159/000448528 27562336

[13] Chua HR Schneider AG Baldwin I Collins A Ho L Bellomo R. Phoxilium vs Hemosol-B0 for continuous renal replacement therapy in acute kidney injury. *J Crit Care* (2013) 28:.e7–14. 10.1016/j.jcrc.2013.02.013 23683569

[14] Godaly G Carlsson O Broman M. Phoxilium(§) reduces hypophosphataemia and magnesium supplementation during continuous renal replacement therapy. *Clin Kidney J.* (2016) 9:205–10. 10.1093/ckj/sfv133 26985370 PMC4792612

[15] Yessayan L Yee J Frinak S Szamosfalvi B. Continuous renal replacement therapy for the management of acid-base and electrolyte imbalances in acute kidney injury. *Adv Chronic Kidney Dis.* (2016) 23:203–10. 10.1053/j.ackd.2016.02.005 27113697

[16] Brooks G. Potassium additive algorithm for use in continuous renal replacement therapy. *Nurs Crit Care.* (2006) 11:273–80. 10.1111/j.1478-5153.2006.00185.x 17883675

[17] Godden J Spexarth F Dahlgren M. Standardization of continuous renal-replacement therapy fluids using a commercial product. *Am J Health Syst Pharm.* (2012) 69:786–93. 10.2146/ajhp110325 22517023

[18] Rewa OG Villeneuve PM Lachance P Eurich DT Stelfox HT Gibney RTN Quality indicators of continuous renal replacement therapy (CRRT) care in critically ill patients: A systematic review. *Intensive Care Med.* (2017) 43:750–63. 10.1007/s00134-016-4579-x 27730284

[19] Tseng J Halbert RJ Minissian N Rodriguez H Barathan S Hain P Association of standardization of continuous renal replacement therapy and high-value care: An evidence-based approach. *JAMA Intern Med.* (2018) 178:572–3. 10.1001/jamainternmed.2017.8732 29482214 PMC5876928

[20] Kovesdy CP Regidor DL Mehrotra R Jing J McAllister CJ Greenland S Serum and dialysate potassium concentrations and survival in hemodialysis patients. *Clin J Am Soc Nephrol.* (2007) 2:999–1007. 10.2215/CJN.04451206 17702709

[21] Pun PH Lehrich RW Honeycutt EF Herzog CA Middleton JP. Modifiable risk factors associated with sudden cardiac arrest within hemodialysis clinics. *Kidney Int.* (2011) 79:218–27. 10.1038/ki.2010.315 20811332

[22] Tiong MK Ullah S McDonald SP Tan SJ Lioufas NM Roberts MA Serum phosphate and mortality in incident dialysis patients in Australia and New Zealand. *Nephrology (Carlton).* (2021) 26:814–23. 10.1111/nep.13904 34046973

[23] Schutte AE Kollias A Stergiou GS. Blood pressure and its variability: Classic and novel measurement techniques. *Nat Rev Cardiol.* (2022):1–12. 10.1038/s41569-022-00690-0 [Epub ahead of print]. 35440738 PMC9017082

[24] Parati G Stergiou GS Dolan E Bilo G. Blood pressure variability: Clinical relevance and application. *J Clin Hypertens (Greenwich).* (2018) 20:1133–7. 10.1111/jch.13304 30003704 PMC8030809

[25] Yang Y Long C Li T Chen Q. Insulin Degludec versus insulin glargine on glycemic variability in diabetic patients: A systematic review and meta-analysis of randomized controlled trials. *Front Endocrinol (Lausanne).* (2022) 13:890090. 10.3389/fendo.2022.890090 35721710 PMC9204495

[26] Agusti AG Torres A Estopa R Agustividal A. Hypophosphatemia as a cause of failed weaning: The importance of metabolic factors. *Crit Care Med.* (1984) 12:142–3. 10.1097/00003246-198402000-00012 6697732

[27] O’Connor LR Wheeler WS Bethune JE. Effect of hypophosphatemia on myocardial performance in man. *N Engl J Med.* (1977) 297:901–3. 10.1056/NEJM197710272971702 904668

[28] Wang L Xiao C Chen L Zhang X Kou Q. Impact of hypophosphatemia on outcome of patients in intensive care unit: A retrospective cohort study. *BMC Anesthesiol.* (2019) 19:86. 10.1186/s12871-019-0746-2 31122196 PMC6533764

[29] Heung M Mueller BA. Prevention of hypophosphatemia during continuous renal replacement therapy-An overlooked problem. *Semin Dial.* (2018) 31:213–8. 10.1111/sdi.12677 29405468

[30] Troyanov S Geadah D Ghannoum M Cardinal J Leblanc M. Phosphate addition to hemodiafiltration solutions during continuous renal replacement therapy. *Intensive Care Med.* (2004) 30:1662–5. 10.1007/s00134-004-2333-2 15156308

[31] Pistolesi V Zeppilli L Polistena F Sacco MI Pierucci A Tritapepe L Preventing continuous renal replacement therapy-induced hypophosphatemia: An extended clinical experience with a phosphate-containing solution in the setting of regional citrate anticoagulation. *Blood Purif.* (2017) 44:8–15. 10.1159/000453443 28219057

[32] Santoro A Mancini E London G Mercadal L Fessy H Perrone B Patients with complex arrhythmias during and after haemodialysis suffer from different regimens of potassium removal. *Nephrol Dial Transplant.* (2008) 23:1415–21. 10.1093/ndt/gfm730 18065796

[33] Severi S Vecchietti S Cavalcanti S Mancini E Santoro A. Electrocardiographic changes during hemodiafiltration with different potassium removal rates. *Blood Purif.* (2003) 21:381–8. 10.1159/000073440 14586180

[34] Hung AM Hakim RM. Dialysate and serum potassium in hemodialysis. *Am J Kidney Dis.* (2015) 66:125–32. 10.1053/j.ajkd.2015.02.322 25828570

[35] Ferrey A You AS Kovesdy CP Nakata T Veliz M Nguyen DV Dialysate potassium and mortality in a prospective hemodialysis cohort. *Am J Nephrol.* (2018) 47:415–23. 10.1159/000489961 29879714 PMC6204129

[36] Besnard N Serveaux M Machado S Daubin D Brunot V Amigues L Electrolytes-enriched hemodiafiltration solutions for continuous renal replacement therapy in acute kidney injury: A crossover study. *Blood Purif.* (2016) 42:18–26. 10.1159/000444248 26949936

[37] DeVita MV Gardenswartz MH Konecky A Zabetakis PM. Incidence and etiology of hyponatremia in an intensive care unit. *Clin Nephrol.* (1990) 34:163–6. 2257702

[38] Polderman KH Schreuder WO Strack van Schijndel RJ Thijs LG. Hypernatremia in the intensive care unit: An indicator of quality of care? *Crit Care Med.* (1999) 27:1105–8. 10.1097/00003246-199906000-00029 10397213

[39] Bagshaw SM Townsend DR McDermid RC. Disorders of sodium and water balance in hospitalized patients. *Can J Anaesth.* (2009) 56:151–67. 10.1007/s12630-008-9017-2 19247764

[40] Dangoisse C Dickie H Tovey L Ostermann M. Correction of hyper- and hyponatraemia during continuous renal replacement therapy. *Nephron Clin Pract.* (2014) 128:394–8. 10.1159/000369347 25592652

[41] Ostermann M Dickie H Tovey L Treacher D. Management of sodium disorders during continuous haemofiltration. *Crit Care.* (2010) 14:418. 10.1186/cc9002 20519032 PMC2911705

[42] Zivin JR Gooley T Zager RA Ryan MJ. Hypocalcemia: A pervasive metabolic abnormality in the critically ill. *Am J Kidney Dis.* (2001) 37:689–98. 10.1016/S0272-6386(01)80116-5 11273867

[43] Sigwalt F Bouteleux A Dambricourt F Asselborn T Moriceau F Rimmelé T. Clinical complications of continuous renal replacement therapy. *Contrib Nephrol.* (2018) 194:109–17. 10.1159/000485608 29597222

[44] Liu C Mao Z Kang H Hu J Zhou F. Regional citrate versus heparin anticoagulation for continuous renal replacement therapy in critically ill patients: A meta-analysis with trial sequential analysis of randomized controlled trials. *Crit Care.* (2016) 20:144. 10.1186/s13054-016-1299-0 27176622 PMC4866420

[45] Himmel HM Whorton AR Strauss HC. Intracellular calcium, currents, and stimulus-response coupling in endothelial cells. *Hypertension.* (1993) 21:112–27. 10.1161/01.HYP.21.1.112 8380279

[46] Smith JB. Calcium homeostasis in smooth muscle cells. *New Horiz.* (1996) 4:2–18. 8689272

[47] Afshinnia F Belanger K Palevsky PM Young EW. Effect of ionized serum calcium on outcomes in acute kidney injury needing renal replacement therapy: Secondary analysis of the acute renal failure trial network study. *Ren Fail.* (2013) 35:1310–8. 10.3109/0886022X.2013.828258 23992422 PMC4082331

[48] Tan HK Bellomo R M’Pisi DA Ronco C. Ionized serum calcium levels during acute renal failure: Intermittent hemodialysis vs. Continuous hemodiafiltration. *Ren Fail.* (2002) 24:19–27. 10.1081/JDI-120002657 11921695

[49] Klein CJ Moser-Veillon PB Schweitzer A Douglass LW Reynolds HN Patterson KY Magnesium, calcium, zinc, and nitrogen loss in trauma patients during continuous renal replacement therapy. *JPEN J Parenter Enteral Nutr.* (2002) 26:77–92. 10.1177/014860710202600277 11871740

[50] Leblanc M Moreno L Robinson OP Tapolyai M Paganini EP. Bicarbonate dialysate for continuous renal replacement therapy in intensive care unit patients with acute renal failure. *Am J Kidney Dis.* (1995) 26:910–7. 10.1016/0272-6386(95)90055-1 7503065

[51] Lemon SJ Zack SD Voils SA. No difference in mechanical ventilation-free hours in critically ill patients who received intravenous, oral, or enteral phosphate replacement. *J Crit Care.* (2017) 39:31–5. 10.1016/j.jcrc.2017.01.002 28152386

[52] Lameire NH Bagga A Cruz D De Maeseneer J Endre Z Kellum JA Acute kidney injury: An increasing global concern. *Lancet.* (2013) 382:170–9. 10.1016/S0140-6736(13)60647-9 23727171

[53] Chertow GM Burdick E Honour M Bonventre JV Bates DW. Acute kidney injury, mortality, length of stay, and costs in hospitalized patients. *J Am Soc Nephrol.* (2005) 16:3365–70. 10.1681/ASN.2004090740 16177006

