# Supplementary material for: Impact of protocolized fluid management on electrolyte stability in patients undergoing continuous renal replacement therapy
Source: Front Med (Lausanne). 2022 Aug 31;9:915072. doi: 10.3389/fmed.2022.915072 (PMC9471083; doi:10.3389/fmed.2022.915072)
Supplement: Supplementary file 1 [file Data_Sheet_1.docx]

Supplementary Material

Impact of protocolized fluid management on electrolyte stability in patients undergoing continuous renal replacement therapy

Song In Baeg, MD^1,2^; Junseok Jeon, MD^2^; Danbee Kang, PhD^3,4^; Soo Jin Na, MD^5^; Juhee Cho, PhD^3,4^; Kyunga Kim, PhD^6^; Jeong Hoon Yang, MD, PhD^5^; Chi Ryang Chung, MD, PhD^5^; Jung Eun Lee, MD, PhD^2^; Wooseong Huh, MD, PhD^2^; Gee Young Suh, MD, PhD^5^; Yoon-Goo Kim, MD, PhD^2^; Dae Joong Kim, MD, PhD^2^; Hye Ryoun Jang, MD, PhD^2^

*^1^Department of Internal Medicine, Division of Nephrology, Myongji Hospital, Hanyang University Medical Center, Goyang, Republic of Korea*

*^2^ Department of Medicine, Division of Nephrology, Samsung Medical Center, Samsung Biomedical Research Institute, Sungkyunkwan University School of Medicine, Seoul, Republic of Korea*

*³ Department of Clinical Research Design and Evaluation, Samsung Advanced Institute for Health Sciences & Technology, Sungkyunkwan University, Seoul, Republic of Korea*

*^4^ Center of Clinical Epidemiology, Samsung Medical Center, Seoul, Republic of Korea*

*^5^ Department of Critical Care Medicine, Samsung Medical Center, Sungkyunkwan University School of Medicine, Seoul, Republic of Korea*

*^6^ Statistics and Data Center, Samsung Medical Center, Seoul, Republic of Korea*

*** Correspondence:**Hye Ryoun Jang, MD, PhD

[shinehr@skku.edu](mailto:shinehr@skku.edu)

***Table S1.*** The composition of CRRT fluid

| Composition (mmol/L) | Hemosol B0 | Phoxilium | MultiBic 4K |
| --- | --- | --- | --- |
| Sodium | 140 | 140 | 140 |
| Potassium | 0 | 4 | 4 |
| Chloride | 109.5 | 116 | 113 |
| Calcium | 1.75 | 1.25 | 1.5 |
| Magnesium | 0.5 | 0.6 | 0.5 |
| Phosphate | 0 | 1.2 | 0 |
| Bicarbonate, mEq/L | 32 | 30 | 35 |
| Lactate | 3 | 0 | 0 |
| Glucose | 0 | 0 | 5.55 |
| Osmolality, mosm/L | 287 | 293 | 300 |

***Abbreviation***: CRRT, continuous renal replacement therapy

***Table S2.*** CRRT fluid protocol according to serum ionized calcium and potassium levels

| Serum K level (mmol/L) | Dialysate | Pre-replacement fluid | Post-replacement fluid |
| --- | --- | --- | --- |
| K ≤ 4.5 | MultiBic 4K | Hemosol B0 without A bag+  1 bag of KCL 20meq/NS 100mL | Hemosol B0 without A bag |
| 4.6 ≤ K ≤ 5.0 | MultiBic 4K | Hemosol B0 without A bag | Hemosol B0 without A bag |
| K ≥ 5.1 | Hemosol B0 | Hemosol B0 without A bag | Hemosol B0 without A bag |

***Abbreviations:*** CRRT, continuous renal replacement therapy; K, potassium (mmol/L)

***Notes:*** This protocol applied to patients who with persistent or progressive hypercalcemia (ionized calcium ≥ 1.45mmol/L) or had underlying disease causing hypercalcemia. A bag of Hemosol B0 contains calcium chloride dihydrate, magnesium chloride hexahydrate and lactic acid. B bag of Hemosol B0 contains sodium bicarbonate and sodium chloride.

***Table S3.*** The levels of total electrolytes during CRRT

| Serum electrolytes | Total  (n=1853) | Pre-protocol (n=955) | Protocol  (n=898) | *p-*value |
| --- | --- | --- | --- | --- |
| P, mg/dL | 3.1 [2.5-3.9] | 3.0 [2.4-3.8] | 3.2 [2.7-3.9] | <0.01 |
| K, mmol/L | 4.0 [3.7-4.4] | 3.8 [3.6-4.2] | 4.2 [3.9-4.5] | <0.01 |
| Na, mmol/L | 136 [134-138] | 135 [133-137] | 137 [135-139] | <0.01 |
| Mg, mg/dL | 2.1 [1.9-2.2] | 2.0 [1.9-2.2] | 2.1 [1.9-2.3] | <0.01 |
| iCa, mmol/L | 1.19 [1.12-1.26] | 1.24 [1.16-1.30] | 1.16 [1.11-1.22] | <0.01 |
| HCO₃, mg/dL | 21.4 [18.5-23.9 | 21.4 [18.1-24.1] | 21.4 [18.9-23.7] | 0.56 |

***Abbreviations:*** HCO₃, bicarbonate; iCa, ionized calcium; K, potassium; Mg, magnesium; Na, sodium; P, phosphate

***Table S4.*** Subgroup analysis for CVs of electrolytes

| Serum electrolytes | Baseline value | Group | Number of case | CV, median [IQR] | *p-*value |
| --- | --- | --- | --- | --- | --- |
| P | Normal range | Total | 715 | 0.221 [0.167-0.280] | - |
|  |  | Pre-protocol | 335 | 0.248 [0.187-0.313] | <0.01 |
|  |  | Protocol | 380 | 0.204 [0.159-0.260] |  |
|  | Abnormal range | Total | 979 | 0.280 [0.203-0.360] | - |
|  |  | Pre-protocol | 482 | 0.306 [0.222-0.396] | <0.01 |
|  |  | Protocol | 497 | 0.252 [0.188-0.333] |  |
| K | Normal range | Total | 1204 | 0.089 [0.068-0.112] | - |
|  |  | Pre-protocol | 592 | 0.096 [0.077-0.120] | <0.01 |
|  |  | Protocol | 612 | 0.079 [0.061-0.101] |  |
|  | Abnormal range | Total | 560 | 0.112 [0.085-0.150] | - |
|  |  | Pre-protocol | 283 | 0.125 [0.095-0.165] | <0.01 |
|  |  | Protocol | 277 | 0.100 [0.076-0.132] |  |
| Na | Normal range | Total | 840 | 0.015[0.011-0.020] | - |
|  |  | Pre-protocol | 394 | 0.016 [0.012-0.021] | <0.01 |
|  |  | Protocol | 446 | 0.014 [0.011-0.018] |  |
|  | Abnormal range | Total | 921 | 0.018 [0.014-0.025] | - |
|  |  | Pre-protocol | 479 | 0.018 [0.014-0.024] | 0.86 |
|  |  | Protocol | 442 | 0.018 [0.013-0.025] |  |
| Mg | Normal range | Total | 946 | 0.094[0.073-0.119] | - |
|  |  | Pre-protocol | 433 | 0.099 [0.079-0.125] | <0.01 |
|  |  | Protocol | 513 | 0.090 [0.068-0.113] |  |
|  | Abnormal range | Total | 601 | 0.109 [0.085-0.139] | - |
|  |  | Pre-protocol | 247 | 0.111 [0.092-0.139] | 0.17 |
|  |  | Protocol | 354 | 0.107 [0.081-0.140] |  |
| iCa | Normal range | Total | 1112 | 0.045 [0.033-0.061] | - |
|  |  | Pre-protocol | 520 | 0.051 [0.039-0.070] | <0.01 |
|  |  | Protocol | 592 | 0.040 [0.029-0.054] |  |
|  | Abnormal range | Total | 508 | 0.058 [0.043-0.081] | - |
|  |  | Pre-protocol | 238 | 0.070 [0.051-0.097] | <0.01 |
|  |  | Protocol | 270 | 0.053 [0.038-0.069] |  |
| HCO₃ | Normal range | Total | 476 | 0.094 [0.069-0.133] | - |
|  |  | Pre-protocol | 247 | 0.094 [0.068-0.132] | 0.85 |
|  |  | Protocol | 229 | 0.094 [0.070-0.134] |  |
|  | Abnormal range | Total | 1315 | 0.134 [0.095-0.189] | - |
|  |  | Pre-protocol | 674 | 0.148 [0.106-0.207] | <0.01 |
|  |  | Protocol | 641 | 0.123 [0.089-0.171] |  |

***Abbreviations:*** CV, coefficient of variation; HCO₃, bicarbonate; iCa, ionized calcium; K, potassium; Mg, magnesium; Na, sodium; P, phosphate

***Notes:*** Phosphate: normal range (2.5–4.5 mg/dL), abnormal range (≤ 2.4 mg/dL, ≥ 4.6 mg/dL)

Potassium: normal range (3.5–5.1 mmol/L), abnormal range (≤ 3.4 mmo/L, ≥ 5.2 mmol/L)

Sodium: normal range (136–145 mmol/L), abnormal range (≤ 135 mmol/L, ≥ 146 mmol/L)

Magnesium: normal range (1.9–2.5 mg/dL), abnormal range (≤ 1.8 mg/dL, ≥ 2.6 mg/dL)

Ionized calcium: normal range (1.05–1.35 mmol/L), abnormal range (≤ 1.04 mmol/L, ≥ 1.36 mmol/L)

Bicarbonate: normal range (≥ 20 mg/dL), abnormal range (< 20 mg/dL)

***Table S5.*** Subgroup analysis for abnormal event rates of each electrolyte

| Serum electrolytes | Baseline value | Group | Number of observation in normal range | Number of observation in abnormal range | Total observation | Abnormal event rate | 95% CI | *p-*value |
| --- | --- | --- | --- | --- | --- | --- | --- | --- |
| P | Normal range | Total | 8676 | 3858 | 12534 | 0.308 | 0.300-0.316 | - |
|  |  | Pre-protocol | 3450 | 2117 | 5567 | 0.380 | 0.368-0.393 | <0.01 |
|  |  | Protocol | 5226 | 1741 | 6967 | 0.250 | 0.240-0.260 |  |
|  | Abnormal range | Total | 10602 | 5930 | 16532 | 0.359 | 0.351-0.366 | - |
|  |  | Pre-protocol | 4344 | 3217 | 7561 | 0.426 | 0.414-0.437 | <0.01 |
|  |  | Protocol | 6258 | 2713 | 8971 | 0.302 | 0.293-0.312 |  |
| K | Normal range | Total | 19732 | 2819 | 22551 | 0.125 | 0.121-0.129 | - |
|  |  | Pre-protocol | 8447 | 2003 | 10450 | 0.192 | 0.184-0.199 | <0.01 |
|  |  | Protocol | 11285 | 816 | 12101 | 0.067 | 0.063-0.072 |  |
|  | Abnormal range | Total | 7983 | 1737 | 9720 | 0.179 | 0.171-0.186 | - |
|  |  | Pre-protocol | 3814 | 1157 | 4971 | 0.233 | 0.221-0.245 | <0.01 |
|  |  | Protocol | 4169 | 580 | 4749 | 0.122 | 0.113-0.131 |  |
| Na | Normal range | Total | 9829 | 5072 | 14901 | 0.340 | 0.333-0.348 | - |
|  |  | Pre-protocol | 3511 | 3058 | 6569 | 0.466 | 0.454-0.478 | <0.01 |
|  |  | Protocol | 6318 | 2014 | 8332 | 0.242 | 0.233-0.251 |  |
|  | Abnormal range | Total | 7993 | 9295 | 17288 | 0.538 | 0.530-0.545 | - |
|  |  | Pre-protocol | 3134 | 5634 | 8768 | 0.643 | 0.633-0.653 | <0.01 |
|  |  | Protocol | 4859 | 3661 | 8520 | 0.430 | 0.419-0.440 |  |
| Mg | Normal range | Total | 12745 | 3372 | 16117 | 0.210 | 0.203-0.216 | - |
|  |  | Pre-protocol | 4871 | 1800 | 6671 | 0.270 | 0.259-0.281 | <0.01 |
|  |  | Protocol | 7874 | 1572 | 9446 | 0.166 | 0.159-0.174 |  |
|  | Abnormal range | Total | 7641 | 2467 | 10108 | 0.244 | 0.236-0.252 | - |
|  |  | Pre-protocol | 2670 | 1211 | 3881 | 0.312 | 0.298-0.327 | <0.01 |
|  |  | Protocol | 4971 | 1256 | 6227 | 0.202 | 0.192-0.212 |  |
| iCa | Normal range | Total | 16835 | 2051 | 18886 | 0.109 | 0.104-0.113 | - |
|  |  | Pre-protocol | 7052 | 1209 | 8261 | 0.146 | 0.139-0.154 | <0.01 |
|  |  | Protocol | 9783 | 842 | 10625 | 0.079 | 0.074-0.084 |  |
|  | Abnormal range | Total | 7147 | 1487 | 8634 | 0.172 | 0.164-0.180 |  |
|  |  | Pre-protocol | 2770 | 821 | 3591 | 0.229 | 0.215-0.242 | <0.01 |
|  |  | Protocol | 4377 | 666 | 5043 | 0.132 | 0.123-0.141 |  |
| HCO₃ | Normal range | Total | 14962 | 4051 | 19013 | 0.213 | 0.207-0.219 | - |
|  |  | Pre-protocol | 8315 | 2236 | 10551 | 0.212 | 0.204-0.220 | 0.68 |
|  |  | Protocol | 6647 | 1815 | 8462 | 0.215 | 0.206-0.223 |  |
|  | Abnormal range | Total | 34350 | 22924 | 57274 | 0.400 | 0.396-0.404 | - |
|  |  | Pre-protocol | 18895 | 13455 | 32350 | 0.416 | 0.411-0.421 | <0.01 |
|  |  | Protocol | 15455 | 9469 | 24924 | 0.380 | 0.374-0.386 |  |

***Abbreviations:*** HCO₃, bicarbonate; iCa, ionized calcium; K, potassium; Mg, magnesium; Na, sodium; P, phosphate

***Notes:*** Phosphate: normal range (2.5–4.5 mg/dL), abnormal range (≤ 2.4 mg/dL, ≥ 4.6 mg/dL)

Potassium: normal range (3.5–5.1 mmol/L), abnormal range (≤ 3.4 mmo/L, ≥ 5.2 mmol/L)

Sodium: normal range (136–145 mmol/L), abnormal range (≤ 135 mmol/L, ≥ 146 mmol/L)

Magnesium: normal range (1.9–2.5 mg/dL), abnormal range (≤ 1.8 mg/dL, ≥ 2.6 mg/dL)

Ionized calcium: normal range (1.05–1.35 mmol/L), abnormal range (≤ 1.04 mmol/L, ≥ 1.36 mmol/L)

Bicarbonate: normal range (≥ 20 mg/dL), abnormal range (< 20 mg/dL)

***Table S6.*** Analysis of hypo-abnormal event rates and hyper-abnormal event rates

| Serum electrolytes | Number of observations | Pre-protocol | Protocol | *p-*value |
| --- | --- | --- | --- | --- |
| P | Hypo | 3835 (29.2%) | 2643 (16.6%) | <0.01 |
|  | Hyper | 1499 (11.4%) | 1811 (11.4%) |  |
|  | Normal | 7794 (59.4%) | 11484 (72.1%) |  |
|  | Total | 13128 | 15938 |  |
| K | Hypo | 2778 (18.0%) | 711 (4.2%) | <0.01 |
|  | Hyper | 382 (2.5%) | 685 (4.1%) |  |
|  | Normal | 12261 (79.5%) | 15454 (91.7%) |  |
|  | Total | 15421 | 16850 |  |
| Na | Hypo | 8407 (54.8%) | 5221 (31.0%) | <0.01 |
|  | Hyper | 285 (1.9%) | 454 (2.7%) |  |
|  | Normal | 6645 (43.3%) | 11177 (66.3%) |  |
|  | Total | 15337 | 16852 |  |
| Mg | Hypo | 2518 (23.9%) | 1781 (11.4%) | <0.01 |
|  | Hyper | 493 (4.7%) | 1047 (6.7%) |  |
|  | Normal | 7541 (71.5%) | 12845 (82.0%) |  |
|  | Total | 10552 | 15673 |  |
| iCa | Hypo | 596 (5.0%) | 1100 (7.0%) | <0.01 |
|  | Hyper | 1434 (12.1%) | 408 (2.6%) |  |
|  | Normal | 9822 (82.9%) | 14160 (90.4%) |  |
|  | Total | 11852 | 15668 |  |

***Abbreviations:*** iCa, ionized calcium; K, potassium; Mg, magnesium; Na, sodium; P, phosphate

Phosphate (hypophosphatemia ≤2.4 mg/dL, hyperphosphatemia ≥4.6 mg/dL)

Potassium (hypokalemia ≤3.4 mmo/L , hyperkalemia ≥5.2 mmol/L)

Sodium (hyponatremia ≤135 mmol/L, hypernatremia ≥146 mmol/L)

Magnesium (hypomagnesemia ≤1.8 mg/dL, hypermagnesemia ≥2.6 mg/dL)

Ionized calcium (hypocalcemia ≤1.04 mmol/L, hypercalcemia ≥1.36 mmol/L)

***Table S7.*** Cost analysis

| Variables | Pre-protocol | Protocol | *p-*value |
| --- | --- | --- | --- |
| Length of CRRT (days) |  |  |  |
| Median (IQR) | 4 (3-7) | 4 (2-6) | 0.08 |
| Ratio (95% CI)* | Reference | 0.94 (0.89, 0.99) | 0.04 |
| CRRT-related cost ($) |  |  |  |
| Total |  |  |  |
| Median (IQR) | 2,012 (1,441 – 3,188) | 1,884 (1,404 – 2,905) | 0.045 |
| Ratio (95% CI)* | Reference | 0.94 (0.89, 0.99) | 0.04 |
| Management cost ($) |  |  |  |
| Median (IQR) | 1,520 (1,108 – 2,216) | 1,314 (1,108 – 1,933) | <0.01 |
| Ratio (95% CI)* | Reference | 0.92 (0.88, 0.97) | <0.01 |
| CRRT fluid cost ($) |  |  |  |
| Median (IQR) | 539 (342 – 961) | 512 (346 – 940) | 0.9 |
| Ratio (95% CI)* | Reference | 1.03 (0.93, 1.13) | 0.60 |
| Replacement cost ($) |  |  |  |
| Median (IQR) | 8 (4 – 15) | 4 (1 – 10) | <0.01 |
| Ratio (95% CI)* | Reference | 0.21 (0.17, 0.27) | <0.01 |

***Note:*** Values in parenthesis are 95% confidence intervals. Ratios were estimated from mixed models with random intercepts with log_e_ (cost + 1) as the outcome and adjusted for age and sex
